# Supplementary material for: Potential factors influencing COVID-19 vaccine acceptance and hesitancy: A systematic review
Source: PLoS One. 2022 Mar 23;17(3):e0265496. doi: 10.1371/journal.pone.0265496 (PMC8942251; doi:10.1371/journal.pone.0265496)
Supplement: S1 Dataset — (DOCX) [file pone.0265496.s002.docx]

| **Author** | **Total palpations (N)** | **Respondents (n)** | **%** | **Safety** | **Country** |
| --- | --- | --- | --- | --- | --- |
| Soares et al., 2021 | 1943 | 1263 | 65 | 1 | Portugal |
| Jain et al., 2021 | 1068 | 113 | 10.6 | 1 | India |
| Lin et al., 2020 | 3541 | 2570 | 72.6 | 1 | United States |
| Wang K et al., 2021 | 1000 | 784 | 78.4 | 1 | China |
| Suresh et al., 2021 | 358 | 29 | 8.1 | 1 | India |
| Abedin et al., 2021 | 3646 | 936 | 25.4 | 1 | Bangladesh |
| Bai et al., 2021 | 2881 | 2289 | 79.45 | 1 | China |
| El-Elimat et al., 2021 | 3100 | 456 | 14.7 | 1 | Jordan |
| Cai et al., 2021 | 1057 | 246 | 23.3 | 1 | China |
| Almaghaslah et al., 2021 | 862 | 337 | 39.1 | 1 | Saudi Arabia |
| Silva et al., 2021 | 237 | 88 | 37 | 1 | United States |
| Manning et al., 2021 | 1029 | 222 | 21.6 | 1 | United States |
| Sharun et al., 2020 | 351 | 158 | 45 | 1 | India |
| Palm et al., 2021 | 1123 | 159 | 14.1 | 1 | United States |
| Pogue et al., 2020 | 316 | 164 | 51.8 | 1 | United States |
| Wang J et al., 2020 | 2058 | 984 | 47.8 | 1 | China |
| Al-Mulla et al., 2021 | 454 | 209 | 46 | 1 | Qatar |
| Kanyike et al., 2021 | 600 | 376 | 62.7 | 1 | Uganda |
| Petravić et al., 2021 | 624 | 209 | 33.5 | 1 | Slovenia |
| Grochowska et al., 2021 | 239 | 23 | 9.6 | 1 | Poland |
| Rosental&Shmueli, 2021 | 628 | 112 | 17.8 | 1 | Israel |
| Jiang et al., 2021 | 1488 | 232 | 15.6 | 1 | China |
| Mudenda et al., 2021 | 326 | 232 | 71.1 | 1 | Zambia |
| Lazarus et al., 2021 | 13426 | 560 | 4.1 | 1 | Multi (19) |
| Faezi et al., 2021 | 1880 | 1135 | 60.4 | 1 | Africa and middle East |
| Nikolovski et al., 2021 | 7402 | 217 | 2.9 | 1 | United States |
| Burhamah et al., 2021 | 2345 | 72 | 3 | 1 | Kuwait |
| Holzmann-Littig et al., 2021 | 4500 | 197 | 4.3 | 1 | Germany |
|  |  |  |  |  |  |
| AVERAGE | 2088.642857 | 513.2857143 | 34.4625 |  |  |
| **Efficacy** | | | | | |
| **Author** | **Total populations (N)** | **Respondents (n)** | **%** | **Efficacy** | **Coutry** |
| Jain et al., 2021 | 1068 | 113 | 10.6 | 1 | India |
| Linet al., 2020 | 3541 | 2521 | 72.2 | 1 | United States |
| Tavolacci et al., 2021 | 3089 | 50 | 1.6 | 1 | France |
| Almaghaslah et al., 2021 | 862 | 337 | 39.1 | 1 | Saudi Arabia |
| Kanyike et al., 2021 | 600 | 376 | 62.7 | 1 | Uganda |
| Kose et al., 2021 | 1138 | 357 | 31.4 | 1 | Turkey |
| Nikolovski et al., 2021 | 7402 | 217 | 2.9 | 1 | United States |
| Freeman et al., 2021 | 5114 | 480 | 9.3 | 1 | United Kingdom |
|  |  |  |  |  |  |
| AVERAGE | 2851.75 | 556.375 | 28.725 |  |  |
| **Information** | | | | | |
| **Author** | **Total populations (N)** | **Respondents (n)** | **%** | **Information** | **Country** |
| Soares et al., 2021 | 1943 | 1263 | 65 | 1 | Portugal |
| Lin et al., 2020 | 3541 | 3261 | 92.1 | 1 | United States |
| Suresh et al., 2021 | 358 | 104 | 29 | 1 | India |
| Almaghaslah et al., 2021 | 862 | 337 | 39.1 | 1 | Saudi Arabia |
| Silva et al., 2021 | 237 | 38 | 16 | 1 | United States |
| Lucia et al., 2020 | 167 | 37 | 22.1 | 1 | United States |
| Kanyike et al., 2021 | 600 | 376 | 62.7 | 1 | Uganda |
| Saied et al., 2021 | 2133 | 1550 | 72.7 | 1 | Egypt |
| Riad et al., 2021 | 6639 | 2091 | 31.5 | 1 | Multi (22) |
| Sherman et al., 2021 | 1500 | 540 | 36 | 1 | United Kingdom |
| Nikolovski et al., 2021 | 7402 | 5590 | 75.5 | 1 | United States |
| Kaplan et al., 2021 | 1574 | 817 | 51.9 | 1 | Turkey |
|  |  |  |  |  |  |
| AVERAGE | 2246.333333 | 1333.666667 | 49.46667 |  |  |
| **Trust** | | | | | |
| **Author** | **Total populations (N)** | **Respondents (n)** | **%** | **Trust** | **Country** |
| Soares et al., 2021 | 1943 | 1263 | 65 | 1 | Portugal |
| Jain et al., 2021 | 1068 | 113 | 10.6 | 1 | India |
| El-Elimat et al., 2021 | 3100 | 561 | 18.1 | 1 | Jordan |
| Lucia et al., 2020 | 167 | 25 | 14.98 | 1 | United States |
| Kanyike et al., 2021 | 600 | 376 | 62.7 | 1 | Uganda |
| Petravić et al., 2021 | 624 | 209 | 33.5 | 1 | Slovenia |
| Riad et al., 2021 | 6639 | 2130 | 32.1 | 1 | Multi (22) |
| Lazarus et al., 2021 | 13426 | 3826 | 28.5 | 1 | Multi (19) |
| Mascarenhas et al., 2021 | 248 | 163 | 65.6 | 1 | United States |
| Kose et al., 2021 | 1138 | 357 | 31.4 | 1 | Turkey |
| Padhi& Al-Mohaithef,2021 | 992 | 70 | 7 | 1 | Saudi Arabia |
| Kelekar et al., 2021 | 415 | 96 | 23 | 1 | United States |
| Grüner&Krüger, 2020 | 208 | 42 | 20.2 |  | Germany |
| Freeman et al., 2021 | 5114 | 179 | 3.5 | 1 | United Kingdom |
| Holzmann-Littig et al., 2021 | 4500 | 115 | 2.5 | 1 | Germany |
|  |  |  |  |  |  |
| AVERAGE | 2678.8 | 635 | 27.912 |  |  |
| **Side Effect** | | | | | |
| **Author** | **Total populations (N)** | **Respondents (n)** | **%** | **Side Effect** | **Country** |
| Suresh et al., 2021 | 358 | 158 | 44.1 | 1 | India |
| Tavolacci et al., 2021 | 3089 | 48 | 1.55 | 1 | France |
| Bai et al., 2021 | 2881 | 2289 | 79.45 | 1 | China |
| El-Elimat et al., 2021 | 3100 | 814 | 26.3 | 1 | Jordan |
| Manning et al., 2021 | 1029 | 222 | 21.6 | 1 | United States |
| Lucia et al., 2020 | 167 | 83 | 49.7 | 1 | United States |
| Kanyike et al., 2021 | 600 | 376 | 62.7 | 1 | Uganda |
| Petravić et al., 2021 | 624 | 209 | 33.5 | 1 | Slovenia |
| Rosental&Shmueli, 2021 | 628 | 112 | 17.8 | 1 | Israel |
| Jiang et al., 2021 | 1488 | 232 | 15.6 | 1 | China |
| Bono et al., 2021 | 10183 | 4195 | 41.2 | 1 | LMICs (9) |
| Saied et al., 2021 | 2133 | 2064 | 96.8 | 1 | Egypt |
| Riad et al., 2021 | 6639 | 3369 | 50.7 | 1 | Multi (22) |
| Mudenda et al., 2021 | 326 | 256 | 78.5 | 1 | Zambia |
| Szmyd et al., 2021 | 1971 | 224 | 11.3 | 1 | Poland |
| Kose et al., 2021 | 1138 | 357 | 31.4 | 1 | Turkey |
| Sherman et al., 2021 | 1500 | 540 | 36 | 1 | United Kingdom |
| Faezi et al., 2021 | 1880 | 1135 | 60.4 | 1 | Africa and middle East |
| Arce et al., 2021 | 15928 | 6371 | 40 | 1 | LMICs (Asia, Africa, South America) |
| Nikolovski et al., 2021 | 7402 | 2770 | 37.4 | 1 | United States |
| Freeman et al., 2021 | 5114 | 480 | 9.3 | 1 | United Kingdom |
| Holzmann-Littig et al., 2021 | 4500 | 302 | 6.7 | 1 | Germany |
|  |  |  |  |  |  |
| AVERAGE | 3303.545455 | 1209.363636 | 38.72727 |  |  |
| **Effectiveness** | | | | | |
| **Athor** | **Total populations (N)** | **Respondents (n)** | **%** | **Effectiveness** | **Country** |
| Wang et al., 2021 | 1000 | 304 | 78.4 | 1 | China |
| Abedin et al., 2021 | 3646 | 936 | 25.4 | 1 | Bangladesh |
| El-Elimat et al., 2021 | 3100 | 456 | 14.7 | 1 | Jordan |
| Almaghaslah et al., 2021 | 862 | 430 | 49.9 | 1 | Saudi Arabia |
| Silva et al., 2021 | 237 | 57 | 24 | 1 | United States |
| Sharun et al., 2020 | 351 | 189 | 53.8 |  | India |
| Palm et al., 2021 | 1123 | 159 | 14.1 | 1 | United States |
| Pogue et al., 2020 | 316 | 34 | 10.65 | 1 | United States |
| Wang J et al., 2020 | 2058 | 1558 | 75.7 | 1 | China |
| Lucia et al., 2020 | 167 | 31 | 18.5 | 1 | United States |
| Al-Mulla et al., 2021 | 454 | 72 | 16 | 1 | Qatar |
| Grochowska et al., 2021 | 239 | 23 | 9.6 | 1 | Poland |
| Bono et al., 2021 | 10183 | 1538 | 15.1 | 1 | LMICs (9) |
| Saied et al., 2021 | 2133 | 1988 | 93.2 | 1 | Egypt |
| Mudenda et al., 2021 | 326 | 33 | 10.2 | 1 | Zambia |
| Lazarus et al., 2021 | 13426 | 560 | 4.1 | 1 | Multi (19) |
| Reiter et al., 2021 | 2006 | 622 | 31 | 1 | United States |
| Nikolovski et al., 2021 | 7402 | 136 | 1.8 | 1 | United States |
| Holzmann-Littig et al., 2021 | 4500 | 540 | 12 | 1 | Germany |
|  |  |  |  |  |  |
| AVERAGE | 2817.315789 | 508.7368421 | 29.37632 |  |  |
| **Cons_Beliefs** | | | | | |
| **Author** | **Total populations (N)** | **Respondents (n)** | **%** | **Cons_Beliefs** | **Country** |
| Pogue et al., 2020 | 316 | 3 | 1 | 1 | United States |
| Lazarus et al., 2021 | 13426 | 1947 | 14.5 | 1 | Multi (19) |
| Szmyd et al., 2021 | 1971 | 310 | 15.7 | 1 | Poland |
| Islam et al., 2021 | 637 | 57 | 9 | 1 | Multi (52) |
| Sallam et al., 2021a | 2173 | 602 | 27.7 | 1 | Jordan |
| Sallam et al., 2021a | 771 | 180 | 23.4 | 1 | Kwait |
| Sallam et al., 2021b | 1106 | 206 | 18.6 | 1 | Jordan |
| Burhamah et al., 2021 | 2345 | 106 | 4.5 | 1 | Kuwait |
|  |  |  |  |  |  |
| AVERAGE | 2843.125 | 426.375 | 14.3 |  |  |
| **Social_Influ** | | | | | |
| **Author** | **Total populations (N)** | **Respondents (n)** | **%** | **Social_Influ** | **Country** |
| Lin et al., 2020 | 3541 | 2914 | 82.3 | 1 | United States |
| Tavolacci et al., 2021 | 3089 | 17 | 0.55 | 1 | France |
| Cai et al., 2021 | 1057 | 332 | 31.4 | 1 | China |
| Mascarenhas et al., 2021 | 248 | 167 | 67.4 | 1 | United States |
| Freeman et al., 2021 | 5114 | 3422 | 67 | 1 | United Kingdom |
| Holzmann-Littig et al., 2021 | 4500 | 183 | 4 | 1 | Germany |
|  |  |  |  |  |  |
| AVERAGE | 2924.833333 | 1172.5 | 42.10833 |  |  |
| **Political role** | | | | | |
| **Author** | **Total populations (N)** | **Respondents (n)** | **%** | **Political role** | **Country** |
| Palm et al., 2021 | 1123 | 158 | 14 | 1 | United States |
| Riad et al., 2021 | 6639 | 1068 | 16 | 1 | Multi (22) |
| Reiter et al., 2021 | 2006 | 653 | 32.5 | 1 | United States |
| Holzmann-Littig et al., 2021 | 4500 | 206 | 4.5 | 1 | Germany |
|  |  |  |  |  |  |
| AVERAGE | 3567 | 521.25 | 16.75 |  |  |
| **Vaccine-mandate** | | | | | |
| **Author** | **Total populations (N)** | **Respondents (n)** | **%** | **Mandate** | **Contry** |
| Almaghaslah et al., 2021 | 862 | 402 | 46.7 | 1 | Saudi Arabia |
| Silva et al., 2021 | 237 | 201 | 85 | 1 | United States |
| Lucia et al., 2020 | 167 | 17 | 10.1 | 1 | United States |
| Mascarenhas et al., 2021 | 248 | 156 | 63 | 1 | United States |
|  |  |  |  |  |  |
| AVERAGE | 378.5 | 194 | 51.2 |  |  |
| **Fear & Anxiety** | | | | | |
| **Author** | **Total populations (N)** | **Respondents (n)** | **%** | **Fear & Anxiety** | **Country** |
| Rosental&Shmueli, 2021 | 628 | 112 | 17.8 | 1 | Israel |
| Nikolovski et al., 2021 | 7402 | 126 | 1.7 | 1 | United States |
| Holzmann-Littig et al., 2021 | 4500 | 302 | 6.7 | 1 | Germany |
| AVERAGE | 4176.66 | 180 | 8.73 |  |  |

| **Asia** | **Safety** | | | | |
| --- | --- | --- | --- | --- | --- |
| **Author** | **Total populations (N)** | **Respondents (n)** | **%** | **Safety** | **Country** |
| Jain et al., 2021 | 1068 | 113 | 10.6 | 1 | India |
| Wang K et al., 2021 | 1000 | 784 | 78.4 | 1 | China |
| Suresh et al., 2021 | 358 | 29 | 8.1 | 1 | India |
| Abedin et al., 2021 | 3646 | 936 | 25.4 | 1 | Bangladesh |
| Bai et al., 2021 | 2881 | 2289 | 79.45 | 1 | China |
| El-Elimat et al., 2021 | 3100 | 456 | 14.7 | 1 | Jordan |
| Cai et al., 2021 | 1057 | 246 | 23.3 | 1 | China |
| Almaghaslah et al., 2021 | 862 | 337 | 39.1 | 1 | Saudi Arabia |
| Sharun et al., 2020 | 351 | 158 | 45 | 1 | India |
| Wang J et al., 2020 | 2058 | 984 | 47.8 | 1 | China |
| Al-Mulla et al., 2021 | 454 | 209 | 46 | 1 | Qatar |
| Rosental&Shmueli, 2021 | 628 | 112 | 17.8 | 1 | Israel |
| Jiang et al., 2021 | 1488 | 232 | 15.6 | 1 | China |
| Burhamah et al., 2021 | 2345 | 72 | 3 | 1 | Kuwait |
| AVERAGE | 1521.142857 | 496.9285714 | 32.44643 |  |  |
|  |  |  |  |  |  |
| **Europe** |  | | | | |
| Soares et al., 2021 | 1943 | 1263 | 65 | 1 | Portugal |
| Petravić et al., 2021 | 624 | 209 | 33.5 | 1 | Slovenia |
| Grochowska et al., 2021 | 239 | 23 | 9.6 | 1 | Poland |
| Holzmann-Littig et al., 2021 | 4500 | 197 | 4.3 | 1 | Germany |
| AVERAGE | 1826.5 | 423 | 28.1 |  |  |
|  |  |  |  |  |  |
| **USA** |  | | | | |
| Lin et al., 2020 | 3541 | 2570 | 72.6 | 1 | United States |
| Silva et al., 2021 | 237 | 88 | 37 | 1 | United States |
| Manning et al., 2021 | 1029 | 222 | 21.6 | 1 | United States |
| Palm et al., 2021 | 1123 | 159 | 14.1 | 1 | United States |
| Pogue et al., 2020 | 316 | 164 | 51.8 | 1 | United States |
| Nikolovski et al., 2021 | 7402 | 217 | 2.9 | 1 | United States |
| AVERAGE | 2274.666667 | 570 | 33.33333 |  |  |
|  |  |  |  |  |  |
| **Africa** |  |  |  |  |  |
| Kanyike et al., 2021 | 600 | 376 | 62.7 | 1 | Uganda |
| Mudenda et al., 2021 | 326 | 232 | 71.1 | 1 | Zambia |
| Faezi et al., 2021 | 1880 | 1135 | 60.4 | 1 | Africa and middle East |
| AVERAGE | 935.3333333 | 581 | 64.73333 |  |  |
|  |  |  |  |  |  |
| **Multi-ethnic** |  |  |  |  |  |
| Lazarus et al., 2021 | 13426 | 560 | 4.1 | 1 | Multi (19) |
|  |  |  |  |  |  |
| **Asia** | **Efficacy** | | | | |
| **Author** | **Total populations (N)** | **Respondents (n)** | **%** | **Efficacy** | **Country** |
| Jain et al., 2021 | 1068 | 113 | 10.6 | 1 | India |
| Almaghaslah et al., 2021 | 862 | 337 | 39.1 | 1 | Saudi Arabia |
| Kose et al., 2021 | 1138 | 357 | 31.4 | 1 | Turkey |
| AVERAGE | 1022.666667 | 269 | 27.03333333 |  |  |
|  |  |  |  |  |  |
| **USA** |  |  |  |  |  |
| Linet al., 2020 | 3541 | 2521 | 72.2 | 1 | United States |
| Nikolovski et al., 2021 | 7402 | 217 | 2.9 | 1 | United States |
| AVERAGE | 5471.5 | 1369 | 37.55 |  |  |
|  |  |  |  |  |  |
| **Europe** |  |  |  |  |  |
| Tavolacci et al., 2021 | 3089 | 50 | 1.6 | 1 | France |
| Freeman et al., 2021 | 5114 | 480 | 9.3 | 1 | United Kingdom |
| AVERAGE | 4101.5 | 265 | 5.45 |  |  |
|  |  |  |  |  |  |
| **Africa** |  |  |  |  |  |
| Kanyike et al., 2021 | 600 | 376 | 62.7 | 1 | Uganda |
|  |  |  |  |  |  |
| **Asia** | **Information** | | | | |
| **Author** | **Total populations (N)** | **Respondents (n)** | **%** | **Information** | **Country** |
| Suresh et al., 2021 | 358 | 104 | 29 | 1 | India |
| Almaghaslah et al., 2021 | 862 | 337 | 39.1 | 1 | Saudi Arabia |
| Kaplan et al., 2021 | 1574 | 817 | 51.9 | 1 | Turkey |
| AVERAGE | 931.3333333 | 419.3333333 | 40 |  |  |
|  |  |  |  |  |  |
| **Europe** |  |  |  |  |  |
| Soares et al., 2021 | 1943 | 1263 | 65 | 1 | Portugal |
| Sherman et al., 2021 | 1500 | 540 | 36 | 1 | United Kingdom |
| AVERAGE | 1721.5 | 901.5 | 50.5 |  |  |
|  |  |  |  |  |  |
| **USA** |  |  |  |  |  |
| Lin et al., 2020 | 3541 | 3261 | 92.1 | 1 | United States |
| Silva et al., 2021 | 237 | 38 | 16 | 1 | United States |
| Lucia et al., 2020 | 167 | 37 | 22.1 | 1 | United States |
| Nikolovski et al., 2021 | 7402 | 5590 | 75.5 | 1 | United States |
| AVERAGE | 2836.75 | 2231.5 | 51.425 |  |  |
|  |  |  |  |  |  |
| **Africa** |  |  |  |  |  |
| Kanyike et al., 2021 | 600 | 376 | 62.7 | 1 | Uganda |
| Saied et al., 2021 | 2133 | 1550 | 72.7 | 1 | Egypt |
| AVERAGE | 1366.5 | 963 | 67.7 |  |  |
|  |  |  |  |  |  |
| **Multi ethnic** |  |  |  |  |  |
| Riad et al., 2021 | 6639 | 2091 | 31.5 | 1 | Multi (22) |
|  |  |  |  |  |  |
| **Asia** | **Trust** | | | | |
| **Author** | **Total populations (N)** | **Respondents (n)** | **%** | **Trust** | **Country** |
| Jain et al., 2021 | 1068 | 113 | 10.6 | 1 | India |
| El-Elimat et al., 2021 | 3100 | 561 | 18.1 | 1 | Jordan |
| Kose et al., 2021 | 1138 | 357 | 31.4 | 1 | Turkey |
| Padhi& Al-Mohaithef,2021 | 992 | 70 | 7 | 1 | Saudi Arabia |
| AVERAGE | 1574.5 | 275.25 | 16.775 |  |  |
|  |  |  |  |  |  |
| **Europe** |  |  |  |  |  |
| Soares et al., 2021 | 1943 | 1263 | 65 | 1 | Portugal |
| Petravić et al., 2021 | 624 | 209 | 33.5 | 1 | Slovenia |
| Grüner&Krüger, 2020 | 208 | 42 | 20.2 |  | Germany |
| Freeman et al., 2021 | 5114 | 179 | 3.5 | 1 | United Kingdom |
| Holzmann-Littig et al., 2021 | 4500 | 115 | 2.5 | 1 | Germany |
| AVERAGE | 2477.8 | 361.6 | 24.94 |  |  |
|  |  |  |  |  |  |
| **USA** |  |  |  |  |  |
| Lucia et al., 2020 | 167 | 25 | 14.98 | 1 | United States |
| Mascarenhas et al., 2021 | 248 | 163 | 65.6 | 1 | United States |
| Kelekar et al., 2021 | 415 | 96 | 23 | 1 | United States |
| AVERAGE | 276.6666667 | 94.66666667 | 34.52666667 |  |  |
|  |  |  |  |  |  |
| **Africa** |  |  |  |  |  |
| Kanyike et al., 2021 | 600 | 376 | 62.7 | 1 | Uganda |
|  |  |  |  |  |  |
| **Multi ethnic** |  |  |  |  |  |
| Riad et al., 2021 | 6639 | 2130 | 32.1 | 1 | Multi (22) |
| Lazarus et al., 2021 | 13426 | 3826 | 28.5 | 1 | Multi (19) |
| AVERAGE | 10032.5 | 2978 | 30.3 |  |  |
|  |  |  |  |  |  |
| **Asia** | **Side Effect** | | | | |
| **Author** | **Total populations (N)** | **Respondents (n)** | **%** | **Side Effect** | **Country** |
| Suresh et al., 2021 | 358 | 158 | 44.1 | 1 | India |
| Bai et al., 2021 | 2881 | 2289 | 79.45 | 1 | China |
| El-Elimat et al., 2021 | 3100 | 814 | 26.3 | 1 | Jordan |
| Rosental&Shmueli, 2021 | 628 | 112 | 17.8 | 1 | Israel |
| Jiang et al., 2021 | 1488 | 232 | 15.6 | 1 | China |
| Kose et al., 2021 | 1138 | 357 | 31.4 | 1 | Turkey |
| AVERAGE | 1598.833333 | 660.3333333 | 35.775 |  |  |
|  |  |  |  |  |  |
| **Europe** |  |  |  |  |  |
| Tavolacci et al., 2021 | 3089 | 48 | 1.55 | 1 | France |
| Petravić et al., 2021 | 624 | 209 | 33.5 | 1 | Slovenia |
| Szmyd et al., 2021 | 1971 | 224 | 11.3 | 1 | Poland |
| Sherman et al., 2021 | 1500 | 540 | 36 | 1 | United Kingdom |
| Freeman et al., 2021 | 5114 | 480 | 9.3 | 1 | United Kingdom |
| Holzmann-Littig et al., 2021 | 4500 | 302 | 6.7 | 1 | Germany |
| AVERAGE | 2799.666667 | 300.5 | 16.39167 |  |  |
|  |  |  |  |  |  |
| **USA** |  | | | | |
| Manning et al., 2021 | 1029 | 222 | 21.6 | 1 | United States |
| Lucia et al., 2020 | 167 | 83 | 49.7 | 1 | United States |
| Nikolovski et al., 2021 | 7402 | 2770 | 37.4 | 1 | United States |
| AVERAGE | 2866 | 1025 | 36.23333 |  |  |
|  |  |  |  |  |  |
| **Africa** |  |  |  |  |  |
| Kanyike et al., 2021 | 600 | 376 | 62.7 | 1 | Uganda |
| Saied et al., 2021 | 2133 | 2064 | 96.8 | 1 | Egypt |
| Mudenda et al., 2021 | 326 | 256 | 78.5 | 1 | Zambia |
| Faezi et al., 2021 | 1880 | 1135 | 60.4 | 1 | Africa and middle East |
| AVERAGE | 1234.75 | 957.75 | 74.6 |  |  |
|  |  |  |  |  |  |
| **LMICs** |  | | | | |
| Bono et al., 2021 | 10183 | 4195 | 41.2 | 1 | LMICs (9) |
| Arce et al., 2021 | 15928 | 6371 | 40 | 1 | LMICs (Asia, Africa, South America) |
| AVERAGE | 13055.5 | 5283 | 40.6 |  |  |
|  |  |  |  |  |  |
| **Multi ethnic** |  |  |  |  |  |
| Riad et al., 2021 | 6639 | 3369 | 50.7 | 1 | Multi (22) |
|  |  |  |  |  |  |
| **Asia** | **Effectiveness** | | | | |
| **Author** | **Total populations (N)** | **Respondents (n)** | **%** | **Effectiveness** | **Country** |
| Wang et al., 2021 | 1000 | 304 | 78.4 | 1 | China |
| Abedin et al., 2021 | 3646 | 936 | 25.4 | 1 | Bangladesh |
| El-Elimat et al., 2021 | 3100 | 456 | 14.7 | 1 | Jordan |
| Almaghaslah et al., 2021 | 862 | 430 | 49.9 | 1 | Saudi Arabia |
| Sharun et al., 2020 | 351 | 189 | 53.8 |  | India |
| Wang J et al., 2020 | 2058 | 1558 | 75.7 | 1 | China |
| Al-Mulla et al., 2021 | 454 | 72 | 16 | 1 | Qatar |
| AVERAGE | 1638.714286 | 563.5714286 | 44.84286 |  |  |
|  |  |  |  |  |  |
| **Europe** |  |  |  |  |  |
| Grochowska et al., 2021 | 239 | 23 | 9.6 | 1 | Poland |
| Holzmann-Littig et al., 2021 | 4500 | 540 | 12 | 1 | Germany |
| AVERAGE | 2369.5 | 281.5 | 10.8 |  |  |
|  |  |  |  |  |  |
| **USA** |  |  |  |  |  |
| Silva et al., 2021 | 237 | 57 | 24 | 1 | United States |
| Palm et al., 2021 | 1123 | 159 | 14.1 | 1 | United States |
| Pogue et al., 2020 | 316 | 34 | 10.65 | 1 | United States |
| Lucia et al., 2020 | 167 | 31 | 18.5 | 1 | United States |
| Reiter et al., 2021 | 2006 | 622 | 31 | 1 | United States |
| Nikolovski et al., 2021 | 7402 | 136 | 1.8 | 1 | United States |
| AVERAGE | 1875.166667 | 173.1666667 | 16.675 |  |  |
|  |  |  |  |  |  |
| **Africa** |  |  |  |  |  |
| Saied et al., 2021 | 2133 | 1988 | 93.2 | 1 | Egypt |
| Mudenda et al., 2021 | 326 | 33 | 10.2 | 1 | Zambia |
| AVERAGE | 1229.5 | 1010.5 | 51.7 |  |  |
|  |  |  |  |  |  |
| **LMICs** |  |  |  |  |  |
| Bono et al., 2021 | 10183 | 1538 | 15.1 | 1 | LMICs (9) |
|  |  |  |  |  |  |
| **Multi ethnic** |  |  |  |  |  |
| Lazarus et al., 2021 | 13426 | 560 | 4.1 | 1 | Multi (19) |
|  |  |  |  |  |  |
| **Asia** | **Cons Beliefs** | | | | |
| **Author** | **Total populations (N)** | **Respondents (n)** | **%** | **Cons Beliefs** | **Country** |
| Sallam et al., 2021 | 2173 | 602 | 27.7 | 1 | Jordan |
| Sallam et al., 2021 | 771 | 180 | 23.4 | 1 | Kwait |
| Sallam et al., 2021b | 1106 | 206 | 18.6 | 1 | Jordan |
| Burhamah et al., 2021 | 2345 | 106 | 4.5 | 1 | Kuwait |
| AVERAGE | 1598.75 | 273.5 | 18.55 |  |  |
|  |  |  |  |  |  |
| **USA** |  |  |  |  |  |
| Pogue et al., 2020 | 316 | 3 | 1 | 1 | United States |
|  |  |  |  |  |  |
| **Europe** |  |  |  |  |  |
| Szmyd et al., 2021 | 1971 | 310 | 15.7 | 1 | Poland |
|  |  |  |  |  |  |
| **Multi ethnic** |  |  |  |  |  |
| Lazarus et al., 2021 | 13426 | 1947 | 14.5 | 1 | Multi (19) |
| Islam et al., 2021 | 637 | 57 | 9 | 1 | Multi (52) |
| AVERAGE | 7031.5 | 1002 | 11.75 |  |  |
|  |  |  |  |  |  |
| **Asia** | **Social_Influ** | | | | |
| **Author** | **Total populations (N)** | **Respondents (n)** | **%** | **Social_Influ** | **Country** |
| Cai et al., 2021 | 1057 | 332 | 31.4 | 1 | China |
|  |  |  |  |  |  |
| **USA** |  |  |  |  |  |
| Lin et al., 2020 | 3541 | 2914 | 82.3 | 1 | United States |
| Mascarenhas et al., 2021 | 248 | 167 | 67.4 | 1 | United States |
| AVERAGE | 1894.5 | 1540.5 | 74.85 |  |  |
|  |  |  |  |  |  |
| **Europe** |  |  |  |  |  |
| Tavolacci et al., 2021 | 3089 | 17 | 0.55 | 1 | France |
| Freeman et al., 2021 | 5114 | 3422 | 67 | 1 | United Kingdom |
| Holzmann-Littig et al., 2021 | 4500 | 183 | 4 | 1 | Germany |
| AVERAGE | 4234.333333 | 1207.333333 | 23.85 |  |  |
|  |  |  |  |  |  |
| **USA** | **Political role** | | | | |
| **Author** | **Total populations (N)** | **Respondents (n)** | **%** | **Political role** | **Country** |
| Palm et al., 2021 | 1123 | 158 | 14 | 1 | United States |
| Reiter et al., 2021 | 2006 | 653 | 32.5 | 1 | United States |
| AVERAGE | 1564.5 | 405.5 | 23.25 |  |  |
|  |  |  |  |  |  |
| **Europe** |  |  |  |  |  |
| Holzmann-Littig et al., 2021 | 4500 | 206 | 4.5 | 1 | Germany |
|  |  |  |  |  |  |
| **Multi ethnic** |  |  |  |  |  |
| Riad et al., 2021 | 6639 | 1068 | 16 | 1 | Multi (22) |
|  |  |  |  |  |  |
| **Asia** | **Mandate** | | | | |
| **Author** | **Total populations (N)** | **Respondents (n)** | **%** | **Mandate** | **Country** |
| Almaghaslah et al., 2021 | 862 | 402 | 46.7 | 1 | Saudi Arabia |
|  |  |  |  |  |  |
| **USA** |  |  |  |  |  |
| Silva et al., 2021 | 237 | 201 | 85 | 1 | United States |
| Lucia et al., 2020 | 167 | 17 | 10.1 | 1 | United States |
| Mascarenhas et al., 2021 | 248 | 156 | 63 | 1 | United States |
| AVERAGE | 217.3333 | 124.6667 | 52.7 |  |  |
|  |  |  |  |  |  |
| **Asia** | **Fear & Anxiety** | | | | |
| **Author** | **Total populations (N)** | **Respondents (n)** | **%** | **Fear & Anxiety** | **Country** |
| Rosental&Shmueli, 2021 | 628 | 112 | 17.8 | 1 | Israel |
|  |  |  |  |  |  |
| **USA** |  |  |  |  |  |
| Nikolovski et al., 2021 | 7402 | 126 | 1.7 | 1 | United States |
|  |  |  |  |  |  |
| **Europe** |  |  |  |  |  |
| Holzmann-Littig et al., 2021 | 4500 | 302 | 6.7 | 1 | Germany |
