# Supplementary material for: Potential factors influencing COVID-19 vaccine acceptance and hesitancy: A systematic review
Source: PLoS One. 2022 Mar 23;17(3):e0265496. doi: 10.1371/journal.pone.0265496 (PMC8942251; doi:10.1371/journal.pone.0265496)
Supplement: Attachment — Submitted filename: Response to Reviewers_PLOS edited.docx [file pone.0265496.s003.docx]

**Dear Editor,**

In response to the anonymous reviewer’s comments we have revised the paper. Please see below, all tasks and revisions taken are shown in the Table. We hope the paper is now adequately developed for possible publication in the PLOS ONE. However, we don’t have any hesitation to undergo further revision, if you need further revision. Thank you once again for provide us with your valuable comments.

Best Regards,

**Professor Dr. Md. Shah Azam**

**Uiversity of Rajshahi**

**Bangladesh.**

| Reviewers | Reviewer comments | Authors response/action | Revisions shown in manuscript |
| --- | --- | --- | --- |
| RW-1 | The article addresses an important and timely question, seeking to synthesize the available evidence related to factors influencing COVID-19 vaccine acceptance and hesitancy. | Thank you for the appreciation. | . |
| RW-1 | Although the need for such integrative work is conspicuous, I believe that this work in its current shape does not fit the standards of PLOS ONE, warranting a major revision. My chief concern is with the subpar quality of writing. The entire text is rife with awkward wordings and unclear formulations, which oftentimes render it difficult to follow the narrative. In this genre of academic text – integrative reviews – clarity of presentation and clear-cut language are of utmost importance. I suggest that the team of authors enlist outside help, perhaps a professional copy-editing service. | In response to the reviewer’ssuggestion, the manuscript has undergone a thorough editing by a reputed professional copy-editing services provider, MDPI. As a proof of copy-editing services, a certificate has been attached as a supplementary file. | Throughout the manuscript |
| RW-1 | Methodologically, I am not convinced that the search protocol described in lines 167-174 of the text would produce a comprehensive body of texts required to answer the questions that the study seeks to answer. It seems redundant to rely on a string of search terms that include “COVID-19 vaccine hesitancy” and additional terms that, in some cases, can lead to getting at preordained factors (e.g., “COVID-19 vaccine hesitancy and religiosity/conspiracy beliefs”), rather than helping the researchers to discover the full range of factors discussed in the literature. | We have revised search terms, as suggested by the reviewer. We have cross-checked the new search terms in the literature search engines and have found the similar studies what were as included in this analysis. | Page-8, line no: 171-174 |
| RW-1 | Furthermore, the choice to include terms that are this specific should be derived from theory and explained in the protocol development section. | The search items we used in this study were adopted from the recently published scholarly articles on COVID-19 vaccine acceptance and hesitancy and mentioned in methodology section | Page-8, line no: 166-171 |
| RW-2 | In the actual context, the paper presents a theoretical and practical importance. It is quite interesting the analysis of the eleven factors which determine the acceptance or hesitancy towards the Covid-19 vaccines. | Thank you for the appreciation. | . |
| RW-2 | However, the paper was hard to be read because the paragraphs were numbered in the beginning. | It is the style of PLOS One to number the paragraphs. May I please request the editor to send the manuscript to this reviewer without numbering? |  |
